# Inequality in dental services: a scoping review on the role of access toward achieving universal health coverage in oral health

**DOI:** 10.1186/s12903-021-01765-z

**Published:** 2021-08-17

**Authors:** Arash Ghanbarzadegan, Madhan Balasubramanian, Liana Luzzi, David Brennan, Peivand Bastani

**Affiliations:** 1 Australian Research Centre for Population Oral Health, Adelaide Dental School, The University of Adelaide, Adelaide, SA 5000 Australia; 2 Faculty of Medicine and Health, The University of Sydney, Sydney, Australia; 3 Health Human Resources Research Centre, Level 5, School of Health Management and Medical Informatics, Shiraz University of Medical Sciences, Ghasrodasht St., Shiraz, Iran

**Keywords:** Disparities, Oral health, High-income countries, Dental care

## Abstract

**Background:**

Improving access to health services is a way towards achieving universal health coverage (UHC) in oral health. The purpose of this review was to map the determinants of access to dental services within a UHC framework.

**Method:**

Scoping review methods were adopted for the review. PUBMED, Scopus, ISI Web of Science and ProQuest were searched for academic literature on determinants of access to dental services in OCED countries. Articles published in the last 20 years were included. No restriction was placed on study methods; only articles in English language were included. Qualitative synthesis was conducted, along with a trend analysis and mapping exercise.

**Result:**

A total of 4320 articles were identified in the initial search; 57 articles were included in the qualitative synthesis. The results indicate 7 main themes as the determinants of access to dental services: family condition, cultural factors, health demands, affordability of services, availability of services, socio-environmental factors, geographical distance. Defined determinants of access to dental services, family condition, cultural factors and geographical access to dental services can fill the population axis of the UHC cube. Health demands and affordability of services fill the gap of financial protection as another axis of the UHC cube and finally, availability of dental services and socio-environmental factors are aligned with the appropriateness of services, the third axis of the UHC cube.

**Conclusion:**

According to the results, family condition and cultural, health demands, affordability and availability of services, social environment, and geographic factors can affect dental health access and equality. Socio-cultural determinations also need to be considered in applied planning. Addressing these factors to improve access to dental services can pave the way for achieving universal health coverage in oral health and should be considered in different levels of policymaking.

**Supplementary Information:**

The online version contains supplementary material available at 10.1186/s12903-021-01765-z.

## Background

All members of the World Health Organization (WHO) are committed towards achieving universal health coverage (UHC) [1]. UHC can be argued as a practical expression of health equity and is reached when individuals and communities can receive the health services they need without facing any financial barriers [2]. UHC would be achievable when people do not face excessive financial contributions to meet their preventive, curative or rehabilitative needs [3]. Although UHC is one of the post-2015 United Nations' Sustainable Development Goals (SDGs) [2, 4], and it has been well addressed in the WHO 2010 report [5], this goal has not yet been achieved in most societies.

**Fig. 1:**
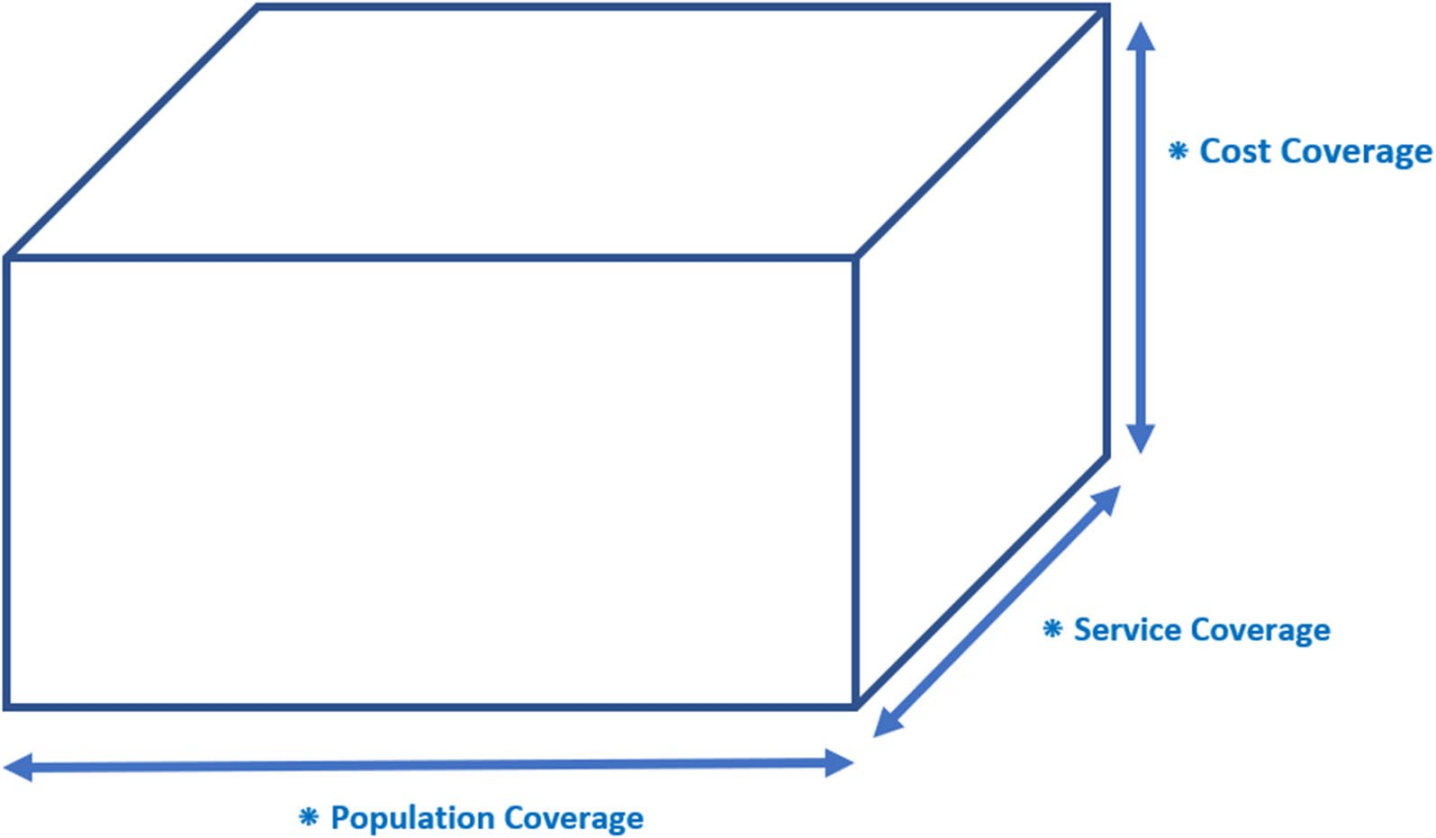
UHC cube [6] adopted from Busse et al

Busse et al. [6] introduced the three dimensions of coverage which are adopted by WHO in their reports [5, 7] to show the way toward UHC and is commonly known as the UHC cube (Fig. 1). According to the cube, these three dimensions of UHC are service, cost and population coverage. To fill this cube and increase universal health, coverage in these three dimensions must improve.

As a separate concept, access to health services refers to the timely use of health services by individuals and the community to achieve the best health outcomes [8]. This concept has different dimensions, including financial access, physical access and acceptability. According to Evans et al. [9], physical accessibility is understood as the availability of good health services within reasonable reach of those who need them and of opening hours, appointment systems and other aspects of service organisation and delivery that allow people to obtain the services when they need them. At the same time, according to Busse et al. [6], if we improve access determinants, we could improve the coverage of services. These two concepts, individuals' acceptability (population coverage) and fair financial access (cost coverage), as two of the three dimensions of the UHC, are reminiscent of the dimensions of access to services Besides those dimensions, service coverage, as the third dimension of the UHC, entails the range of services provided by the health systems. Therefore, it may be argued that in order to achieve the UHC, the dimensions of access should be reached as a proxy for the dimension of the UHC in the populations [9, 10].

At the same time, Campbell et al. [11] have referred to the accessibility of the services as a combination of physical or geographic access, affordability and availability. According to their framework, accessibility of the health services is an important dimension that can affect the quality of the services [11]. In rapid incorporation of the concept from different aforementioned authors, it seems that in spite of different defined components and determinants of access, previous knowledge has emphasised the relationship of access to health services and better performance of health systems toward quality, equality and UHC. So synthesising an overall and comprehensive viewpoint can be fruitful for the health policymakers and health intervention designers.

In addition, although oral health is one of the forgotten levels in macro health policies setting, its importance should not be overlooked [12]. According to Mathur et al. [13], despite the important role of oral health in the community's overall health, well-being, and quality of life, this area has not been adequately considered in many global health plans and strategies. Fisher et al. [14] emphasised the importance of oral diseases as comorbidity factors for sustainable development goals (SDGs) that aim to reduce the burden of diseases and to improve maternal, child and sexual health. Approximately 4.6% of the total health expenditure in the world is for the treatment of oral diseases [15]. Between 3 and 4 billion people suffer from untreated decayed teeth. It should be noted that oral diseases are the most common diseases that human beings suffer from. Oral diseases are among the hundred diseases that cause the most disability-adjusted life years (DALY), and it is even more than the average lost years for a moderate heart failure. Approximately 224 years per 100,000 lives are lost due to disability caused by oral diseases [16].

Oral health and access to dental services should be acknowledged similarly to other health services under UHC to reduce the existing inequalities in the populations. It has been reported that the elimination of financial hardship is not enough to achieve a sustainable improvement in equity unless other factors like geographical access are considered [17]. However, in the dental literature, most studies have addressed physical and geographical access to services which is only one aspect of UHC cube and access to services. Therefore, it is essential to conduct a focused review to determine all dimensions and determinants of access to dental services.

Inequality in dental services has been reported in all societies, including high-income countries, but no framework addressing determinants of access-related inequalities in dental services has yet been reported. Challenges to accessing dental services are seen in most countries, although these challenges are greater in low- and middle-income countries. There are more inconsistencies and knowledge gaps in oral policies in these countries [13, 18]. At the same time, other evidence has implied that socioeconomic factors may affect the style and prevalence of oral and dental challenges in a different way. For instance, a strong negative association has been considered between sugar consumption and caries (B = – 2.80, R2 = 0.17) in both high and low-income countries. Similarly, a strong positive relationship has been reported between DMFT and per capita sugar consumption (B = – 0.89, R2 = 0.20) in high and low-income countries but not among the middle-income ones [19]. These pieces of evidence can highlight the need for attention to this area among high-income countries, as well as the others.

On the other hand, many countries of the Organization for Economic Co-operation and Development (OECD) have adopted different strategies to increase access to dental services that emphasise the need for these countries to manage and improve equality in this area [20]. Among them, we can refer to increasing teledentistry, including dentistry services in insurance packages, supporting rural, remote or tribal areas [21], provision of oral and dental services by physicians and dentists, revising dental and medical schools’ curricula, improving the collaborations among dentists and physicians and so on [22]. Thus, to more accurately investigate this inequality in access to dental services, this study aims to examine the determinants of these access-related inequalities in the OECD countries to date (August 2020). Additionally, this study aims to map the determinants of access-related inequality in dental services to help researchers and policymakers to synthesise the relevant evidence and enable evidence-based policymaking. This map is not only a summary of the access-related inequalities in dental services of OECD countries but also can be a supportive piece of evidence for evidence-based policymaking in other countries.

## Methods

A scoping review methodology was applied to allow the inclusion of a variety of relevant and heterogeneous literature simultaneously. Scoping reviews enable determining the main aspects of a concept and making a comprehensive map of evidence [23]. In this regard, Joanna Briggs Institute (JBI) methodology for scoping reviews was adopted. The JBI scoping review guideline has considered three different approaches in conducting a scoping review; a six-step approach of Arksey and O’Malley [24], that of Levac et al. [26] and the nine-step approach of Peter et al. [25]. We have followed Levac, Colquhoun and O'Brien approach [26]. Table 1 shows the review steps and clarifies the Levac interpretation of the Arkey and O’Mally framework as well.

**Table 1:** The six-step approach of Levac et al. [26] for a scoping review

Step (JBI) [25]	Description (Levac et al.)
Identifying the research question	Clarifying the review propose and linking the purpose and research question
Identifying relevant studies	Feasibility balancing with breadth and comprehensiveness of the scoping process
Study selection	Using an iterative team approach for study selection and data extraction
Charting the data	Including a numerical summary and qualitative thematic analysis
Collating, summarising, and reporting the results	Identifying the implications of the study findings for policy, practise or research
Consultation (optional)	Adopting consultation as a required component of scoping study methodology

**Fig. 2:**
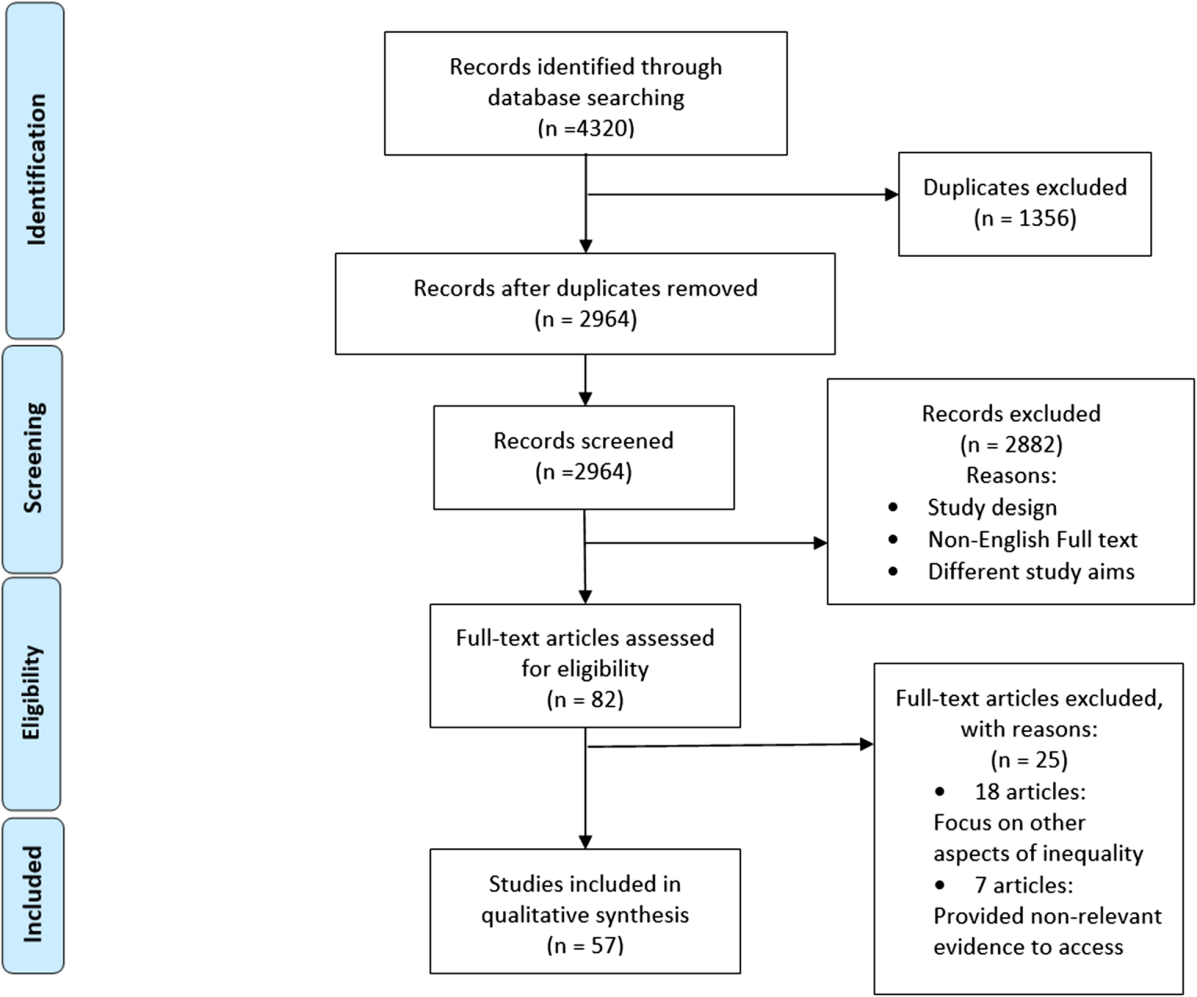
PRISMA flow chart

According to Table 1, The three components of the study design are as follows:

1. Identifying research question, search strategy, data extraction
  This main component has included the initial three steps of Table 1. These three steps are considered as follows:
2. Clarifying and linking the purpose and research question
  The study aimed to explore the main determinants of access-related inequalities in dental services among the OECD countries. In this regard, the main research question was defined as: What are the main determinants affecting access to dental services in the OECD countries?
3. Feasibility balancing with the comprehensiveness of the scoping process
  In this step, the relevant keywords were selected to set the search strategy. The systematic search had been conducted from 01/01/2000 up to 08/08/2020 in 4 scientific databases, including PUBMED, Scopus, ISI Web of Science and ProQuest (Additional file 1: Table S1). Systematic management of the retrieved studies was done using the EndNote reference manager X9 (Clarivate Analytics, Philadelphia, PA, USA).
4. Using an iterative team approach to select studies and extract data
  Inclusion and exclusion criteria were defined. Original articles with different methodological designs (quantitative, qualitative or mixed-method studies) which were available in English full-text and fulfilled the study purpose, were included. On the other hand, review articles (unless reviewing, analysing and reporting a national survey), conference proceedings, editorials and commentaries were excluded from the study. Articles were first screened based on their titles and abstracts, then the full texts of the retrieved abstracts were thoroughly reviewed by two research team members (AG and PB) separately. At the time of any disagreement between the reviewers for including the material, the third person (DB) studied the paper and made the final decision. The Preferred Reporting Items for Systematic Reviews and Meta-Analyses (PRISMA) was applied for illustrating the approach (Fig. 2) [27].
5. Thematic analysis and Mapping exercise (towards UHC themes)
  This component has contained the final two steps of Table 1 as follows:
6. Incorporating a numerical summary and qualitative thematic analysis
  A data extraction form (Additional file 1: Table S2) was applied to record the authors’ name, place of the studies, publication year, and the study’s main finding. Subsequently, thematic analysis was utilised to analyse and synthesise the data by one researcher who has more reflexivity with qualitative research (PB).
7. Identifying the implications of the study findings for policy, practise or research
  The Graneheim and Lundman [28] approach was applied through the following steps: first, extracted data were reviewed several times, initial codes were made and then labelled. Secondly, all initial codes were reviewed and merged to reach the final codes according to the aim of the study. At the third step, final codes were categorised to achieve the sub-themes and the main themes of access to dental services in the OECD countries. Finally, all sub-themes and main themes were tabulated and discussed among the research team to investigate the implications for policymakers. At the same time, data synthesis was conducted from two perspectives: 1. matching the thematic analysis findings with the dimensions of access to services 2. aligning the defined themes with the dimensions of the UHC cube [7].
8. Trend analysis
  This scoping review also utilises trend analysis to illustrate the trends of publications categorised in each of the emerging determinants. To investigate the publication trend of determinants and dimensions of access to dental services, the number of articles in each section was determined, and the relevant bar chart was drawn using Microsoft Excel Version 16 for MAC (Microsoft Corporation, Redmont, WA).

## Results

Results of the study are presented in two sections: first, the description of the characteristics of the included studies and then the results of the thematic analysis and the thematic map.

### Characteristics of the included studies

According to the present search strategy (Additional file 1: Table S1), a total of n = 4320 studies were identified in the title, abstract and keyword search. Following the exclusion of duplicates (n = 1356), a total of 2964 records were screened. 82 articles were selected for full-text reading, and 57 articles included in the final qualitative synthesis (see Fig. 2—PRISMA flowchart). The last 25 articles were omitted for two particular reasons: no evidence relevant to access to dental services (7 articles) and/or aiming for other aspects of inequality such as utilisation and provision rather than access (18 articles).

The characteristics of the included articles showed that most of the studies were conducted in the United States of America (20 articles). Sorting the included studies by the publication year also revealed that most of the studies had been published in the last decade (40 articles). More information about these patterns is available in the Additional file 1: Figures S1 and S2.

### Thematic analysis

Thematic analysis of the included studies led to 7 main determinants of access-related inequality in dental services as follows: family condition factors, health demands, cultural factors, affordability of services, availability of services, socio-environmental factors and geographical access (Table 2). Included articles are mentioned in an additional column in the Additional file 1: Table S3.

**Table 2:** Determinants of access to dental services

Dimensions of access	Main determinants	Sub determinants
Acceptability of services	Family condition	Existence of an elderly member in the family
		Existence of a child in the family
		Families living in poverty
		Race/ethnic minority/aboriginality of a family
		Occurring pregnancy in the family
		Member living alone
		Education level of the whole family
		Primary language spoken at home
		Number of children at the shelter
	Cultural factors	Fear of dental treatment or phobias
		Oral health beliefs
		Victimisation
		Poor oral health behaviours
Financial	Health demands	Unmet oral healthcare needs
		Health problems
		Poor oral condition
	Affordability of services	Income
		Health insurance
		Cost of services (out of pocket payment)
		Medicaid and medicare
		Federal government’s funding
Physical	Availability of services	Oral health delivery system
		Public coverage of dental services
		Dentists visits/preventive care
		Specialised treatment
		Virtual dental home
		Long waiting time
		High proportion of dentists
		Shelter based care
		Access to oral hygiene products
		Pensioners
	Socio-environmental factors	Refugees
		Immigrants
		Disadvantages people
	Geographical distance	Geographical access
		Travel time
		Using public transportation
		Rural populations
		Living in census areas
		Living in the regions outside major cities

Considering the 3 main dimensions of access to health services; financial, physical and acceptability of the services, Table 2 depicts that the family condition factors and cultural factors can affect the acceptability of access to dental services.

As one of those determinants, family condition factors consist of different subthemes such as being a child, an elder or pregnant family member [29, 30], race or aboriginality [31–33] of the family. Each of the aforementioned factors can have a negative or positive effect on access to dental health services. In other words, according to the included studies, those families with a larger size, those who belong to an the ethnic minority or involve aboriginality and families with a lower educational level along with those who live alone or with a different primary spoken language at home, may have lower access to dental health services while the existence of the elderly, a pregnant mother or having a child at home may intensify the access to dental services particularly at the time of benefiting supportive health benefit packages.

Besides family condition, culture and attitudes toward oral health beliefs and behaviours can raise access related inequality in dental services [34].

Health demands along with the affordability of the services by the patients are aligned with the financial dimension of the access to dental services. Health demands, in the reviewed literature, mainly emphasises the population’s oral health and dental needs. Therefore, society's unmet needs can impose a high financial burden on access to dental services [35] and should be considered along with the revealed needs and the real demands.

Family income [36] or insurance coverage [37, 38] enhance the affordability of the services, particularly those social and basic health benefit packages with a wide coverage of dental services, while out of pocket payment at the time of the need for services may negatively impact access to dental services [39, 40].

Availability of dental services, living or environmental conditions and geographical access are aligned with the physical dimension of access to dental services. Dental visit waiting-time [41] and applying virtual services and telehealth [38, 42] may affect the availability of services and, consequently, access dental services in different ways. Geographical access, travelling time [43] and public transportation utilisation [44] are other determinants of physical access.

In sum, these three dimensions of access; financial, physical and acceptability of services, along with their determinants, are considering as the main dimensions and the sub-dimensions of access to dental services, which have influenced the inequality in terms of access to dental services in the OECD countries (Table 2).

**Fig. 3:**
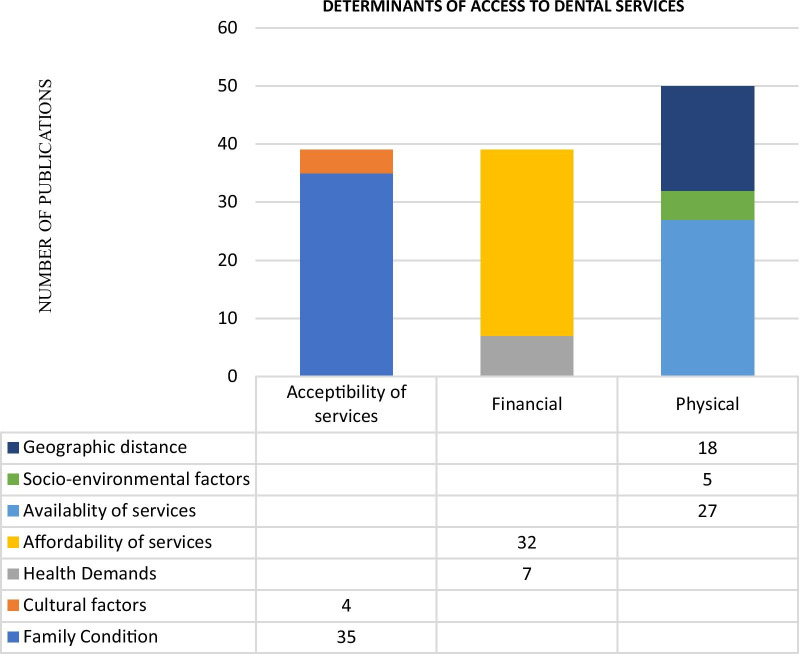
Number of the articles in different dimensions and determinants of access to dental services

Trend analysis of the publications showed that the most published dimension of access to dental services is the physical dimension. Regarding the determinants, most studies covered the affordability of services (32 articles), while a small proportion covered cultural factors (4 articles) and socio-environmental factors (5 articles) (Fig. 3).

Besides the aforementioned synthesis, the evidence can also be aligned with the UHC dimensions. In this regard, family condition, culture, and geographical access to dental services can fill the population aspect of the UHC cube, which extends the coverage toward the population. Socio-environmental determinants and affordability fill another axis of the UHC cube, financial protection, which is similar to the financial dimension of access to services. Financial protection is mainly emphasised on the power of the health system for progressive and equitable funding of the services by allocated taxed or insurance premiums rather than out of pocket (OOP) payments. The potentiality of the health system also influences the affordability of the dental services to provide appropriate services to the community with affordable prices. This factor and the socio-environmental status of the people representing their financial status, insurance status, and power of paying OOP can be considered Financial protection of the UHC cube.

**Fig. 4:**
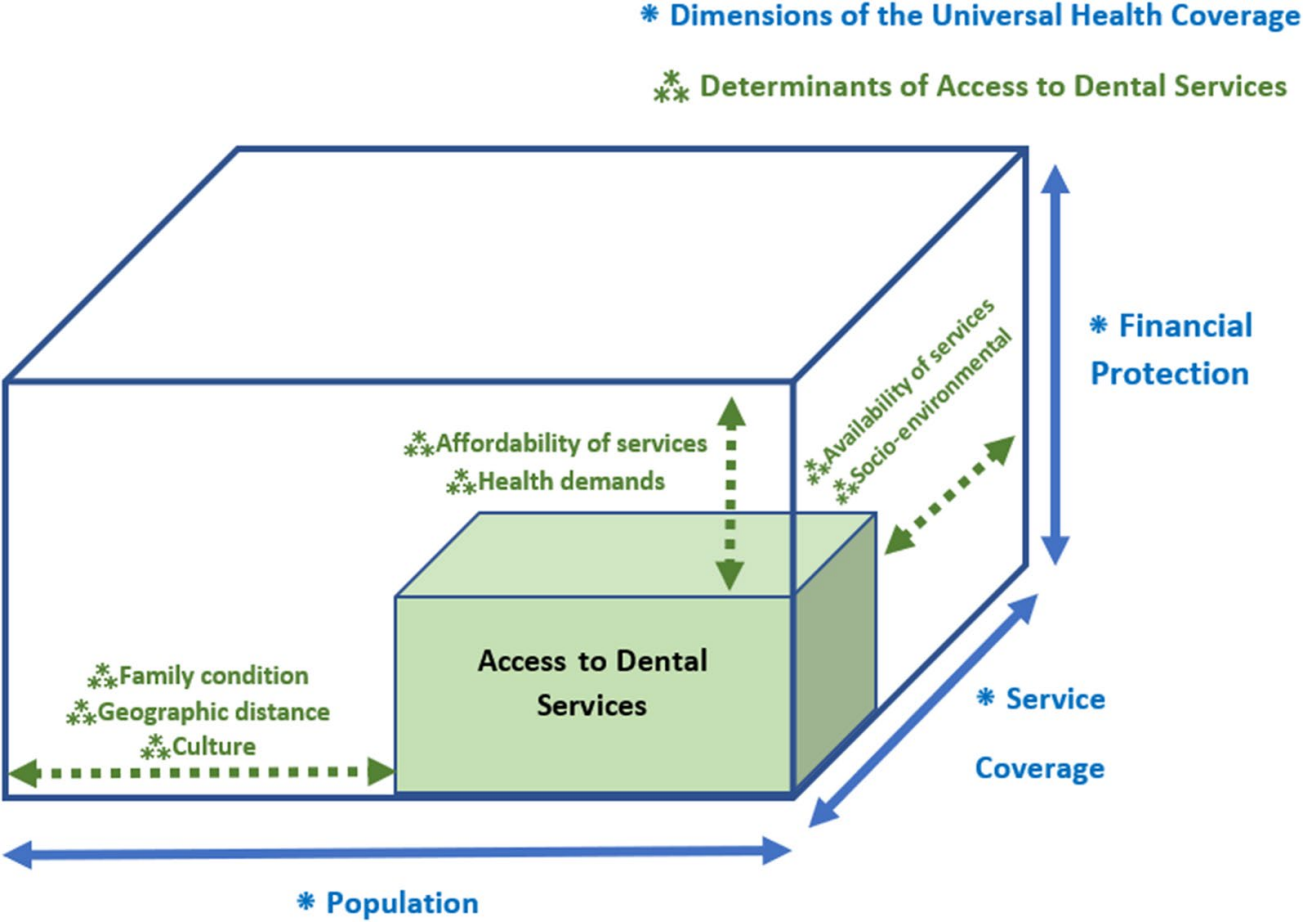
Determinants of access to dental services aligned with the previously introduced Busse et al. cube [6] which was modified as the WHO's UHC cube [7]

Service coverage as the third axis of the UHC cube defines how extensively a health system covers different services. This dimension can be adjusted by the availability of dental services and the individual's health status (Fig. 4).

## Discussion

In this study, we discussed different determinants of access to dental services among OECD countries. According to the current results, access to dental services includes a vast dimension of physical access, financial access, and acceptability.

Physical access is divided into the categories of availability of services, social environment and geographic and transportation regarding the current results. Similarly, according to the literature, geographical access to dental services is only one determinant of access to services [45]. Although geographical access is the clearest determinant, this factor cannot solely determine the population's access to services. It would be significant for policymakers to pay adequate attention to other factors such as culture and other family conditions. These three factors together can be highly influential in determining the rate of access and utilisation to oral health services. At the same time, affordability of the services can significantly affect the dimension of access which emphasised access of all populations without any financial hardship. This factor, and the community’s health demands can shed light for policymakers to plan for increasing access to oral health services and universal health coverage. Finally, the concept of UHC has made the coverage of high-quality services as another prerequisite. According to the current results, policymakers should be sensitive to the availability of oral services for all populations, such as those in rural and remote areas.

Although many OECD countries are at a high individual and national level of wealth and income, even among these countries, income distribution is not fair [46]. This inequality can affect people's access to dental services. Some examples of income inequalities in accessing dental services at the ecological and individual level are as follows: a study in Norway found that counties with higher revenue collections also had higher access rates to dental centres [47]; while when considering individual income, various studies have shown that people with lower income had a lower rate of affordability of services [36, 38, 48].

To increase the affordability of dental services, it is not sufficient to pay attention only to people’s income. Another determinant that can improve this dimension of access to dental services is being covered by insurance. Holding any type of health insurance, not just dental insurance can reduce health expenditures and thus increase the ability of people to pay for dental expenses [41, 49]. However, Newacheck et al. [50] stated that even after insurance expansion for adolescents, disparities remained. Such a discussion can be justified considering that dental services coverage may lead to a restricted scope of care via an insurance scheme enrollment. These schemes can present different facilities and access to services that depends on the government funds, patients’ premiums and the nature of the private or public sector scheme [51]. Therefore, to achieve a desirable outcome, all determinants should be addressed, and at the same time, it should not be neglected that the association and relevance of insurance as a determinant of access is needed to be considered according to the context of universal, public care systems.

Finally, family condition and cultural factors affect access to services in terms of the acceptability of services. Many studies addressed specific family and individual circumstances, such as age, pregnancy and education [30, 52, 53]. While, within the family condition factors, race and ethnicity have been the most studied determinants.

In addition to racial factors, cultural determinants should be considered to increase the acceptability of services. In the included studies, the least attention was paid to these factors, while victimisation and oral health attitudes can affect the level of access to services [34]. This existing gap can be addressed by taking into consideration cultural factors, as well as stigma and social isolation in different social groups.

In sum, inequality in dental health services can be caused as a result of inappropriate access to such services. At the same time, lack of physical and financial access and also the acceptability of dental services can deepen the inequality, particularly among those families with lower socio-cultural and income level. The role of public benefit packages the same as insurance coverage, should not be neglected [54], and both revealed, and the unmet needs of the population should be considered by policymakers to be assured of restricting the inequality by increasing the access to dental services.

Despite the discussion about the dimensions of access and the UHC cube, according to Sanders et al. [51] there is need for further attention to Social Determinants of Health (SDH) and the concepts of community participation and advocacy via UHC. It should not be forgotten that some basic concepts, the same as PHC can be merged with UHC and facilitate the context of better oral and general health for the community. All these concepts should be considered in a comprehensive approach by policymakers depending on the countries context for moving forward towards better access to dental services. Besides considering inequalities in access to dental services, to eliminate inequalities, other aspects of the "triangle of inequalities in dental services" such as inequalities in utilisation and provision of dental services, must be considered [55].  

## Conclusions

According to the results, family condition and cultural factors, health demands, affordability and availability of services, social environment, and geographic factors can affect dental health access and equality. Socio-cultural determinants also need to be considered in applied planning. Addressing these factors to improve access to dental services can pave the way for achieving universal health coverage in Oral Health and should be considered in different levels of policymaking.

## Strengths and limitations

As a focused scoping review requires a homogeneous population, we have limited this study to the OECD countries. Since access determinants to dental services in low- and middle-income countries can be different from the findings of this study, studying that context is also recommended. On the other hand, many the comprehensive studies conducted in OECD countries along with a separate review of two members of the research team who are familiar with the methodology, increased the robustness of the findings. In this study, due to the limitation in translating non-English texts, studies in languages other than English were excluded, which was one of the limitations of this project. We have tried to reduce the selection bias by conducting a scoping review that is more comprehensive than the other types of systematic reviews. However, this bias is an inevitable part of a review, so we encourage researchers to consider this. Distinguishing between the concepts of access and utilisation of services and mapping the identified determinants of access with the UHC dimensions was a novel aspect of this study and could help researchers and policymakers and thus be effective for the community.

## Supplementary Information

**Additional file 1**. Search strategy syntax and results.

## Data Availability

The datasets used and/or analysed during the current study are available from the corresponding author on reasonable request.

## Abbreviations

UHC: Universal health coverage
OECD: Organisation for Economic Co-operation and Development
SDG: Sustainable development goals
WHO: World Health Organization
DALY: Disability-adjusted life year
