# Supplementary material for: Inequality in dental services: a scoping review on the role of access toward achieving universal health coverage in oral health
Source: BMC Oral Health. 2021 Aug 17;21:404. doi: 10.1186/s12903-021-01765-z (PMC8369795; doi:10.1186/s12903-021-01765-z)
Supplement: Supplementary file 1 — Additional file 1. Search strategy syntax and results. [file 12903_2021_1765_MOESM1_ESM.docx]

| **Table S1- The final search strategy of the scoping review**   \| Databases \| PubMed, ProQuest, ISI Web of Science, Scopus \| \| \| \| --- \| --- \| --- \| --- \| \| **Limitations** \| **Time limits** \| \| 01.01.2000 to 08.08.2020 \| \| **Language** \| \| English \| \| **General Search Strategy** \| (#1 AND #2 AND #3) [Title/Abstract] \| \| \| \| #1 \| “Dental Health Surveys” OR “Oral Health Disparities” OR “Dental Health” OR “Oral Health” \| \| \| #2 \| “Socioeconomic Factors” OR “Social Hierarchy” OR “Inequalities” OR “Social Disparities” OR “Social Gradient*” OR “Health Status*” OR “socioeconomic disadvantage” OR “socioeconomic inequalities” OR “Social Determinants” OR “Socio Economic Status” \| \| \| #3 \| ("Dental services" OR "Dental visits" OR "Access") \| \| \| **PubMed** \| ((("Dental Health Surveys"[Mesh]) OR ( "Oral Health"[Mesh] OR ( "Dental Care"[Mesh] OR "Dental Health Services"[Mesh] )) AND ((((("Socioeconomic Factors"[Mesh]) OR "Hierarchy, Social"[Mesh]) OR ( "Healthcare Disparities"[Mesh] OR "Health Status Disparities"[Mesh] )) OR "Social Determinants of Health"[Mesh]) OR "Social Class"[Mesh])) AND (((("dental services"[Title/Abstract]) OR ("dental visits"[Title/Abstract])) OR ("access" [Title/Abstract])) \| \| \| \| **Scopus** \| TITLE-ABS-KEY("oral health") OR TITLE-ABS-KEY("Dental Health Surveys") OR TITLE-ABS-KEY("Dental Health Services") OR TITLE-ABS-KEY("dental care") AND TITLE-ABS-KEY("Socioeconomic Factors") OR TITLE-ABS-KEY("Social Hierarchy") OR TITLE-ABS-KEY(Inequalities) OR TITLE-ABS-KEY("Social Disparities") OR TITLE-ABS-KEY("Social Gradient") OR TITLE-ABS-KEY("Health Status") OR TITLE-ABS-KEY("socioeconomic disadvantage") OR TITLE-ABS-KEY("socioeconomic inequalities") OR TITLE-ABS-KEY("Social Determinants") AND TITLE-ABS-KEY("dental services") OR TITLE-ABS-KEY("dental visits") OR TITLE-ABS-KEY("access") \| \| \| \| **Web of Science** \| TS= ("Dental Health Surveys" OR "Dental Care" OR "Oral Health" OR "Dental Health Services")  AND  TS= ("Socioeconomic Factors" OR "Hierarchy, Social" OR "Healthcare Disparities" OR "Health Status Disparities" OR "Social Determinants of Health" OR "Social Class")  AND  TS= ("dental services" OR "dental visits" OR "access") \| \| \| \| **ProQuest** \| noft(("Dental Health Surveys" OR "Dental Care" OR "Oral Health" OR "Dental Health Services")) AND noft(("Socioeconomic Factors" OR "Hierarchy, Social" OR "Healthcare Disparities" OR "Health Status Disparities" OR "Social Determinants of Health" OR "Social Class") ) AND noft(("dental services" OR "dental visits" OR "Access")) \| \| \|   **Table S2- Data extraction form of the scoping review** | | | | | | | |
| --- | --- | --- | --- | --- | --- | --- | --- | --- | --- | --- | --- | --- | --- | --- | --- | --- | --- | --- | --- | --- | --- | --- | --- | --- | --- | --- | --- | --- | --- | --- | --- | --- | --- | --- | --- | --- | --- | --- | --- | --- | --- | --- | --- | --- | --- | --- | --- |
| **No** | **Author** | **Study Title/aim** | **Study population** | **Year** | **Study Design** | **Study place** | **Key Determinants of the study**  **(in the scopes of utilization, access, provision, equality)** |
|  |  |  |  |  |  |  | - A - B - C - … |
|  |  |  |  |  |  |  |  |

| **Table S3- Determinants of access to dental services** | | | | |
| --- | --- | --- | --- | --- |
| **Dimensions of access** | **Main determinants** | **Sub determinants** | **Number of articles** | **References** |
| Acceptability | Family condition | Existence of an elderly member in the family | 6 | (1-6) |
|  |  | Existence of a child in the family | 10 | (5, 7-15) |
|  |  | Families living in poverty | 1 | (16) |
|  |  | Race / ethnic minority /Aboriginality of the family | 11 | (6, 17-26) |
|  |  | Occurring Pregnancy in the family | 2 | (27, 28) |
|  |  | Member living alone | 1 | (2) |
|  |  | Education level of the whole family | 1 | (20) |
|  |  | Primary language spoken | 2 | (5, 19) |
|  |  | Number of children at the shelter | 1 | (17) |
|  | Culture | Fear of dental treatment or phobias | 1 | (5) |
|  |  | Oral health beliefs | 1 | (17) |
|  |  | Victimization | 1 | (17) |
|  |  | Poor oral health behaviors | 1 | (2) |
| Financial | Health demands | Unmet oral healthcare needs | 4 | (21, 29-31) |
|  |  | Health problems | 1 | (32) |
|  |  | Poor oral condition | 2 | (2, 31) |
|  | Affordability of services | Income | 14 | (1, 2, 10, 11, 15, 18, 20, 21, 32-37) |
|  |  | Health insurance | 13 | (4, 11, 13, 16, 18, 21, 31, 33, 34, 36, 38-40) |
|  |  | Cost of services (Out of pocket payment) | 2 | (33, 41) |
|  |  | Medicaid and Medicare | 1 | (10) |
|  |  | Federal government`s funding | 2 | (14, 42) |
| Physical | Availability of services | Oral health delivery system | 5 | (15, 30, 34, 43, 44) |
|  |  | Public coverage of dental services | 4 | (31, 39, 44, 45) |
|  |  | Dentists visits/Preventive care | 6 | (4, 23, 33, 38, 46, 47) |
|  |  | Specialized treatment | 1 | (7) |
|  |  | Virtual dental home | 1 | (10) |
|  |  | Long waiting time | 3 | (15, 38, 48) |
|  |  | High proportion of dentists | 3 | (11, 23, 49) |
|  |  | Shelter based care | 1 | (17) |
|  |  | Access to oral hygiene products | 2 | (4, 19) |
|  |  | Pensioners | 1 | (15) |
|  | Socio-environmental factors | Refugees | 1 | (48) |
|  |  | Immigrants | 2 | (6, 21) |
|  |  | Disadvantages people | 2 | (15, 23) |
|  | Geographical distance | Geographic access | 12 | (21, 24, 25, 43, 50-57) |
|  |  | Travelling time | 1 | (13) |
|  |  | Using public transportation | 1 | (1) |
|  |  | Rural populations | 2 | (19, 21) |
|  |  | Living in census areas | 1 | (23) |
|  |  | Living in the regions outside major cities | 1 | (1) |

Figure S1- Number of Published Studies by country


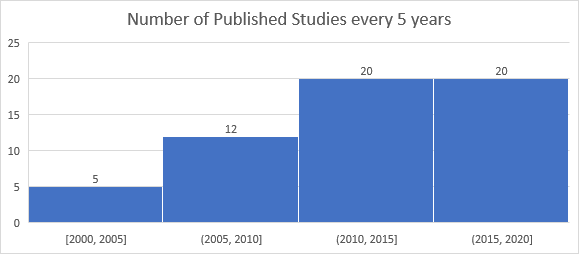


Figure S2- Number of Published Studies every 5 years

**References**

1. Kiuchi S, Aida J, Kusama T, Yamamoto T, Hoshi M, Yamamoto T, et al. Does public transportation reduce inequalities in access to dental care among older adults? Japan Gerontological Evaluation Study. Community Dent Oral Epidemiol. 2020;48(2):109-18.

2. Lee YS, Kim HG, Hur JY, Yang K. Oral Health in Low-Income Older Adults in Korea. J Community Health Nurs. 2016;33(2):98-106.

3. Listl S. Income-related inequalities in dental service utilization by Europeans aged 50+. J Dent Res. 2011;90(6):717-23.

4. Dounis G, Ditmyer MM, McCants R, Lee Y, Mobley C. Southern Nevada assisted living residents' perception of their oral health status and access to dental care. Gerodontology. 2012;29(2):e150-4.

5. Council on Access P, Interprofessional R, Robinson LA. Private sector response to improving oral health care access. Dent Clin North Am. 2009;53(3):523-35.

6. Shelley D, Russell S, Parikh NS, Fahs M. Ethnic disparities in self-reported oral health status and access to care among older adults in NYC. J Urban Health. 2011;88(4):651-62.

7. Morris E, Landes D. The equity of access to orthodontic dental care for children in the North East of England. Public Health. 2006;120(4):359-63.

8. Pinilla J, Negrin-Hernandez MA, Abasolo I. Time trends in socio-economic inequalities in the lack of access to dental services among children in Spain 1987-2011. Int J Equity Health. 2015;14:9.

9. Ravaghi V, Hargreaves DS, Morris AJ. Persistent Socioeconomic Inequality in Child Dental Caries in England despite Equal Attendance. JDR Clin Trans Res. 2020;5(2):185-94.

10. Bailit H, D'Adamo J. State case studies: improving access to dental care for the underserved. J Public Health Dent. 2012;72(3):221-34.

11. Biordi DL, Heitzer M, Mundy E, DiMarco M, Thacker S, Taylor E, et al. Improving access and provision of preventive oral health care for very young, poor, and low-income children through a new interdisciplinary partnership. Am J Public Health. 2015;105 Suppl 2:e23-9.

12. Caban-Martinez AJ, Lee DJ, Fleming LE, Arheart KL, Leblanc WG, Chung-Bridges K, et al. Dental care access and unmet dental care needs among U.S. workers: the National Health Interview Survey, 1997 to 2003. J Am Dent Assoc. 2007;138(2):227-30.

13. Jean G, Kruger E, Tennant M. Universal access to oral health care for Australian children: comparison of travel times to public dental services at consecutive census dates as an indicator of progressive realisation. Aust J Prim Health. 2020;26(2):109-16.

14. Leck V, Randall GE. The rise and fall of dental therapy in Canada: a policy analysis and assessment of equity of access to oral health care for Inuit and First Nations communities. Int J Equity Health. 2017;16(1):131.

15. Schwarz E. Access to oral health care–an Australian perspective. Community Dentistry and Oral Epidemiology. 2006;34(3):225-31.

16. Duncan L, Bonner A. Effects of income and dental insurance coverage on need for dental care in Canada. J Can Dent Assoc. 2014;80:e6.

17. DiMarco MA, Ludington SM, Menke EM. Access to and utilization of oral health care by homeless children/families. Journal of Health Care for the Poor and Underserved. 2010;21(2):67-81.

18. Edelstein BL. Disparities in oral health and access to care: findings of national surveys. Ambulatory pediatrics. 2002;2(2):141-7.

19. Fulkerson ND, Haff DR, Chino M. Health care access disparities among children entering kindergarten in Nevada. J Child Health Care. 2013;17(3):253-63.

20. Lebrun LA, Shi L. Nativity status and access to care in Canada and the U.S.: factoring in the roles of race/ethnicity and socioeconomic status. J Health Care Poor Underserved. 2011;22(3):1075-100.

21. Northridge ME, Kumar A, Kaur R. Disparities in Access to Oral Health Care. Annu Rev Public Health. 2020;41:513-35.

22. Shi L, Lebrun LA, Tsai J. Access to medical care, dental care, and prescription drugs: the roles of race/ethnicity, health insurance, and income. Southern medical journal. 2010;103(6):509.

23. Wamala S, Merlo J, Bostrom G. Inequity in access to dental care services explains current socioeconomic disparities in oral health: the Swedish National Surveys of Public Health 2004-2005. J Epidemiol Community Health. 2006;60(12):1027-33.

24. Jean G, Kruger E, Tennant M. The distribution of allied dental practitioners in australia: socio-economics and rurality as a driver of better health service accessibility. Aust Dent J. 2019;64(2):153-60.

25. Martin-Kerry JM, Whelan M, Rogers J, Raichur A, Cole D, de Silva AM. Addressing disparities in oral disease in Aboriginal people in Victoria: where to focus preventive programs. Australian Journal of Primary Health. 2019;25(4):317-24.

26. Kilpatrick NM, Neumann A, Lucas N, Chapman J, Nicholson JM. Oral health inequalities in a national sample of Australian children aged 2-3 and 6-7 years. Aust Dent J. 2012;57(1):38-44.

27. Carrion IV, Castaneda H, Martinez-Tyson D, Kline N. Barriers impeding access to primary oral health care among farmworker families in Central Florida. Soc Work Health Care. 2011;50(10):828-44.

28. Hunter LP, Yount SM. Oral health and oral health care practices among low-income pregnant women. J Midwifery Womens Health. 2011;56(2):103-9.

29. Calzon Fernandez S, Fernandez Ajuria A, Martin JJ, Murphy MJ. The impact of the economic crisis on unmet dental care needs in Spain. J Epidemiol Community Health. 2015;69(9):880-5.

30. Crete P, Boyd LD, Fitzgerald JK, LaSpina LM. Access to preventive oral health services for homebound populations: A pilot program. American Dental Hygienists' Association. 2018;92(6):24-32.

31. Jones E, Shi L, Hayashi AS, Sharma R, Daly C, Ngo-Metzger Q. Access to oral health care: the role of federally qualified health centers in addressing disparities and expanding access. Am J Public Health. 2013;103(3):488-93.

32. Abbas H, Aida J, Saito M, Tsakos G, Watt RG, Koyama S, et al. Income or education, which has a stronger association with dental implant use in elderly people in Japan? Int Dent J. 2019;69(6):454-62.

33. Howard JR, Ramirez J, Li Y, Gany F. Dental care access for low-income and immigrant cancer patients in New York City. J Community Health. 2015;40(1):110-5.

34. Lupi-Pegurier L, Clerc-Urmes I, Abu-Zaineh M, Paraponaris A, Ventelou B. Density of dental practitioners and access to dental care for the elderly: a multilevel analysis with a view on socio-economic inequality. Health Policy. 2011;103(2-3):160-7.

35. Nishide A, Fujita M, Sato Y, Nagashima K, Takahashi S, Hata A. Income-Related Inequalities in Access to Dental Care Services in Japan. Int J Environ Res Public Health. 2017;14(5).

36. Ramraj C, Sadeghi L, Lawrence HP, Dempster L, Quinonez C. Is accessing dental care becoming more difficult? Evidence from Canada's middle-income population. PLoS One. 2013;8(2):e57377.

37. Guessous I, Theler J-M, Izart CD, Stringhini S, Bodenmann P, Gaspoz J-M, et al. Forgoing dental care for economic reasons in Switzerland: a six-year cross-sectional population-based study. BMC Oral Health. 2014;14(1):121.

38. Jang Y-E, Kim C-B, Kim N-H. Utilization of preventive dental services before and after health insurance covered dental scaling in Korea: 2009 to 2014 Community Health Survey. Asia Pacific Journal of Public Health. 2017;29(1):70-80.

39. Teusner DN, Brennan DS, Spencer AJ. Associations between level of private dental insurance cover and favourable dental visiting by household income. Aust Dent J. 2015;60(4):479-89.

40. Newacheck PW, Hung YY, Jane Park M, Brindis CD, Irwin Jr CE. Disparities in adolescent health and health care: does socioeconomic status matter? Health services research. 2003;38(5):1235-52.

41. Abdus S, Decker SL. Association between Medicaid adult nonemergency dental benefits and dental services use and expenditures. J Am Dent Assoc. 2019;150(1):24-33.

42. Grytten J, Lund E, Rongen G. Equity in access to public dental services: the experience from Norway. Acta Odontologica Scandinavica. 2001;59(6):372-8.

43. Christensen LB, Petersen PE, Steding‐Jessen M. Consumption of dental services among adults in Denmark 1994–2003. European journal of oral sciences. 2007;115(3):174-9.

44. Gallego F, Larroulet C, Palomer L, Repetto A, Verdugo D. Socioeconomic inequalities in self-perceived oral health among adults in Chile. Int J Equity Health. 2017;16(1):23.

45. Kim ES, Kim BI, Jung HI. Does the national dental scaling policy reduce inequalities in dental scaling usage? A population-based quasi-experimental study. BMC Oral Health. 2019;19(1):185.

46. Everaars B, Jerkovic-Cosic K, van der Putten GJ, Pretty IA, Brocklehurst P. Needs in Service Provision for Oral Health Care in Older People: A Comparison Between Greater Manchester (United Kingdom) and Utrecht (the Netherlands). Int J Health Serv. 2018;48(4):663-84.

47. Grignon M, Hurley J, Wang L, Allin S. Inequity in a market-based health system: Evidence from Canada's dental sector. Health Policy. 2010;98(1):81-90.

48. Davidson N, Skull S, Calache H, Chesters D, Chalmers J. Equitable access to dental care for an at‐risk group: a review of services for Australian refugees. Australian and New Zealand journal of public health. 2007;31(1):73-80.

49. Moles D, Frost C, Grundy C. Inequalities in availability of National Health Service general dental practitioners in England and Wales. British dental journal. 2001;190(10):548-53.

50. Armfield JM. Socioeconomic inequalities in child oral health: a comparison of discrete and composite area‐based measures. Journal of Public Health Dentistry. 2007;67(2):119-25.

51. Carta G, Cagetti M, Sale S, Congiu G, Strohmenger L, Oleari F, et al. Oral health inequalities in Italian schoolchildren-a cross-sectional evaluation. Community Dent Health. 2014;31(2):123-8.

52. Chattopadhyay A. Oral health disparities in the United States. Dent Clin North Am. 2008;52(2):297-318, vi.

53. Christensen LB, Rosing K, Lempert SM, Hede B. Patterns of dental services and factors that influence dental services among 64-65-year-old regular users of dental care in Denmark. Gerodontology. 2016;33(1):79-88.

54. Lee H, Seo S, Kang R, Kim Y, Hyun HK. Increasing access to oral healthcare for marriage-immigrant women in South Korea: programme design to policy recommendation. Int Dent J. 2019;69(5):354-60.

55. Piotrowska DE, Jankowska D, Huzarska D, Szpak AS, Pedzinski B. Socioeconomic inequalities in use and non-use of dental services in Poland. Int J Public Health. 2020.

56. Piotrowska DE, Pedzinski B, Jankowska D, Huzarska D, Charkiewicz AE, Szpak AS. Socio-economic inequalities in the use of dental care in urban and rural areas in Poland. Ann Agric Environ Med. 2018;25(3):512-6.

57. Tickle M, Moulding G, Milsom K, Blinkhorn A. Socioeconomic and geographical influences on primary dental care preferences in a population of young children. British dental journal. 2000;188(10):559-62.
